# Serological evidence of *Francisella tularensis* in febrile patients seeking treatment at remote hospitals, northeastern Kenya, 2014–2015

**DOI:** 10.1016/j.nmni.2017.05.015

**Published:** 2017-06-03

**Authors:** J. Njeru, H. Tomaso, K. Mertens, K. Henning, G. Wareth, R. Heller, S. Kariuki, E.M. Fèvre, H. Neubauer, M.W. Pletz

**Affiliations:** 1) Institute of Bacterial Infections and Zoonoses, Friedrich-Loeffler-Institute, Jena, Germany; 2) Centre for Infectious Diseases and Infection Control, Jena University Hospital, Jena, Germany; 3) Centre for Microbiology Research, Kenya Medical Research Institute, Nairobi, Kenya; 4) Institute for Molecular Cell Biology, Center for Molecular Biomedicine, Friedrich Schiller University of Jena, Jena, Germany; 5) Institute of Infection and Global Health, University of Liverpool, Liverpool, UK; 6) International Livestock Research Institute, Nairobi, Kenya; 7) Faculty of Veterinary Medicine, Benha University, Moshtohor, Egypt

**Keywords:** Fever, *Francisella tularensis*, Kenya, seroprevalence, zoonoses

## Abstract

Tularaemia is a highly contagious infectious zoonosis caused by the bacterial agent *Francisella tularensis*. The aim of this study was to investigate the presence of antibodies to *F. tularensis* in febrile patients in northeastern Kenya. During 2014–2015, 730 patients were screened for anti-*F. tularensis* antibodies using a combination of ELISA and Western blot. Twenty-seven (3.7%) individuals were positive for *F. tularensis*. Tularaemia was not suspected by the treating clinicians in any of them. Our results suggest that tularaemia may be present in Kenya but remain unreported, and emphasizes the need for local clinicians to broaden their diagnostic repertoire when evaluating patients with undifferentiated febrile illness.

## Introduction

Tularaemia is a serious zoonotic disease caused by the highly virulent Gram-negative bacterium *Francisella tularensis* [1]. There are two clinically relevant subspecies*: F. tularensis* subspecies *tularensis* and *F. tularensis* subspecies *holarctica. Francisella tularensis tularensis* causes life-threatening tularaemia and is found in North America, whereas *F. tularensis holarctica* causes less-severe tularaemia and is described mainly from the northern hemisphere [2]. Human infection often occurs after inhalation of contaminated dust or after handling infected animals or their products. Tularaemia is also acquired by consumption of contaminated water or raw meat or through bites by infected vectors, i.e. tabanids, ticks or mosquitoes. Many wild animals can be a reservoir of the pathogen [3]. In humans, tularaemia is characterized by fever, chills, fatigue, malaise, headache and lymphadenopathy. Depending upon the route of infection, the clinical forms of tularaemia include ulceroglandular, glandular, oropharyngeal, oculoglandular, typhoidal and pneumonia [4]. Diagnosis is commonly confirmed by serological methods because they are usually easy to perform and cause little risk of infection for laboratory personnel. However, some patients may not seroconvert, while others stay seropositive for years after an infection, and/or may have cross-reacting antibodies due to infections with other Gram-negative bacteria such as *Yersinia* spp., Coxiella spp. and *Brucella* spp. [5], [6]. Because bacteriological isolation of this fastidious organism requires special nutrient media, and is dangerous for laboratory personnel [4], real-time PCR assays have been developed and are becoming widely accepted as alternative and sensitive tools for the diagnosis of acute tularaemia in humans. Additionally, recent multiplex real-time PCR assays have also been developed for identification and discrimination of *F. tularensis* subspecies. The assays have demonstrated high analytical specificity against bacterial pathogens considered to be serologically and genetically related to *F. tularensis* [7], [8], [9], [10].

The epidemiology of tularaemia in Africa remains unknown. A PubMed search using the MeSH terms ‘*Francisella*’ or ‘tularaemia’ and ‘Africa’ revealed only a single case report of *F. tularensis* bacteraemia from Sudan [11]. Although *F. tularensis* has not been documented in Kenya, the previously unrecognized zoonoses including rickettsiosis and Q-fever [12], [13], chikungunya [14], leptospirosis and brucellosis [15], [16] are emerging as common causes of non-malaria febrile illness in Kenya. Such acute bacterial zoonoses show a wide spectrum of clinical symptoms that mimic those from malaria. Due to lack of reliable laboratory diagnostic facilities and the presumptive clinical management of fever as malaria in Kenya [17], [18], many patients with acute zoonotic infections such as tularaemia are likely to be misdiagnosed. This may result in missed opportunities to detect other causes of fever and in routine disease underreporting. We therefore hypothesized that tularaemia is likely to be unreported in Kenya possibly because of low incidence or misdiagnosis for other endemic tropical diseases. The study reported here is part of a broader investigation aimed at advancing our knowledge of the aetiologies of undifferentiated fever in Kenya.

## Materials and methods

### Study participants and sample collection

As part of a comprehensive study of the aetiologies of undifferentiated fever in northeastern Kenya [12], [16], we enrolled patients with acute febrile diseases in Garissa and Wajir hospitals from 2014 through 2015. This province was selected because public health support and disease surveillance have been extremely limited and outbreaks of re-emerging diseases have been reported [15], [19], [20]. The study design was described in detail elsewhere [12]. Briefly, patients presenting with acute febrile illness to the outpatient departments of the two hospitals were systematically enrolled. The inclusion criteria were: a clinical history of acute febrile illness characterized by fever (≥38°C) and at least one of the following clinical symptoms: headache, chills, myalgia, arthralgia, general body malaise and acute lower respiratory illness. The last of these was defined as new onset of cough, difficulties when breathing or chest pain and fever [21]. A study clinician then collected demographic information, obtained clinical history and performed a physical examination of the enrolled patients. Serum samples were obtained from each consenting patient and frozen at –40°C until analysed.

### Detection of anti-*F. tularensis* antibodies

Serum samples were screened for tularaemia using a commercial Serazym^®^ anti-*F. tularensis* ELISA kit (Seramun Diagnostica, Heidesee OT Wolzig, Germany) and results were interpreted according to the manufacturer's instructions. Briefly, samples with an optical density >0.25 were scored as positive while those with optical density <0.20 were considered negative. The samples with values between 0.20 and 0.25 were denoted as borderline. The cut-off values were calculated on the basis of the standard curve corrected by the mean of the extinction of the standard serum according to the manufacturers' instructions. The Serazym Anti-*F. tularensis* ELISA allows detection of all classes of antibodies (IgG, IgA and IgM) to the lipopolysaccharide of *F. tularensis* in human serum.

The samples that gave equivocal results were re-tested.

All serum samples giving positive results by ELISA were further confirmed for anti-*Francisella* lipopolysaccharide-specific antibodies by Western blot with minor modifications to the tests described previously [22]. In our assays, alkaline-phosphatase-labelled goat anti-human IgG (Sigma-Aldrich, Munich, Germany) diluted 1: 30 000 was used as secondary antibody. Sera were considered positive when the *F. tularensis* typical lipopolysaccharide ladder was visible.

To exclude possible cross-reacting antibodies, serum samples were tested for anti-*Coxiella* and anti-*Brucella* antibodies using Serion ELISA classic kits (Virion∖Serion, Würzburg, Germany), and anti-*Yersinia* antibodies using the recomLine *Yersinia* kits (Mikrogen, Neuried, Germany). The results were then interpreted according to the manufacturer's instructions.

### Molecular testing

DNA was extracted from serum samples using High Pure Template preparation Kit™ (Roche Diagnostics, Mannheim, Germany) according to the manufacturer's instructions. Quantitative real-time PCR assays for detection of *Coxiella burnetii* and *Brucella* spp. were performed targeting the *C. burnetii*-specific repetitive element IS1111 and *Brucella* genus-specific bcsp31 gene loci, respectively. The *Brucella* species were identified using the species-specific localization of IS711 element in *Brucella abortus* and *Brucella melitensis* as previously described [23], [24], [25].

### Data management and statistical analysis

Demographic, clinical and serological data were entered and managed by SPSS Statistics software (v.20, IBM Corp.; Armonk, NY, USA). The Pearson's *χ*^2^ test or Fisher's exact test was used to determine differences in tularaemia seroprevalence among demographic groups. Statistical significance was set at p < 0.05.

### Ethics and consent to participate

The approval for collection of specimens from humans was obtained from the Scientific and Ethics Review Unit of Kenya Medical Research Institute, protocol no. 2637. Permission to conduct the study was also sought from relevant public health offices and hospital administration. Written informed consent was obtained from every participant or from guardians of patients <18 years old before specimen collection.

## Results

A total of 1067 patients participated in the overall study including 936 (87.7%) adults (≥18 years). Females accounted for 54.4% of the study population (*n* = 580). Characteristics of the study population have been reported elsewhere [12], [16]. Blood samples of 730 (68.4%) patients were available for tularaemia testing including 652 (69.7%) adults and 78 (59.5%) children.

Overall, 71 (9.7%) patients were seropositive for tularaemia by ELISA, but only 27/730 (3.7%) could be confirmed by Western blotting. Of these, 19/395 (4.8%) were female and 8/335 (2.4%) were male. Of the 71 sera, two were confirmed to be positive for *B. abortus*-specific DNA, and two were seropositive for brucellosis by ELISA only. One patient each tested positive for *C. burnetii* and *Yersinia* spp., respectively. Twenty-seven patients (that were found positive using the combined ELISA and Western blot diagnostic approach), tested negative in all other tests applied.

Analyses of patient characteristics showed no significant associations with tularaemia seropositivity between ethnic groups, sex, residence site or across different age strata, education status and occupations (p > 0.05). The most common clinical presentation (with the exception of constitutional symptoms) among tularaemia-positive patients was lymphadenopathy (59.3%), generalized body aches (55.6%), malaise/fatigue (51.8%) and sore throat (33.3%).

The mean interval from the onset of fever symptoms until hospital presentation was 17.8 days (median 6 days) for tularaemia-positive patients and 15.1 days (median 8 days) for the seronegative group. The symptoms and clinical features of the patients are summarized in Table 1.

**Table 1:** Selected clinical characteristics and numbers seropositive for tularaemia among the study participants, northeastern Kenya^a^

Clinical symptoms and signs	Positive for tularaemia (*N* = 27)	Negative for tularaemia (*N* = 703)	p value
	*n* (%)	*n* (%)	
Lymphadenopathy	16 (59.3)	128 (18.2)	<0.001*
Arthralgia/myalgia	15 (55.6)	506 (72.0)	0.202
Malaise/fatigue	14 (51.9)	499 (70.9)	0.033*
Painful lymph nodes	8 (29.6)	89 (12.8)	0.018*
Headache	8 (29.6)	423 (60.1)	0.080
Weight loss	7 (25.9)	102 (14.5)	0.317
Acute lower respiratory illness	5 (18.5)	152 (21.6)	0.460
Cutaneous lesions	5 (18.5)	111 (15.8)	0.698
Sore throat	9 (33.3)	138 (19.6)	0.109
Vomiting	2 (7.4)	28 (3.9)	0.920
Abdominal pain	4 (14.8)	165 (23.5)	0.064
Diarrhoea	3 (11.1)	87 (12.3)	0.294
Rash	1 (3.7)	35 (4.8)	0.571
Hepato-splenomegaly	3 (11.1)	94 (12.9)	0.610
History of fever (>14 days)	9 (33.3)	262 (37.3)	0.678
Age, mean ± SD (years)	33.2 ± 12.4	34.5 ± 12.1	0.595
Fever onset/median (days)	17.8/6	15.1/8	0.374

*Statistically significant, χ^2^ tests p < 0.05.

a Patients with ELISA-positive results confirmed by Western blot.

Eight of these patients reported painful lymph nodes whereas one patient presented with unilateral suppurative cervical lymphadenopathy. The lymphadenopathies were localized in the inguinal, axillary and cervical regions. Painful lymph node (p = 0.018), malaise or fatigue (p = 0.033) and lymphadenopathy (p < 0.001) were the clinical features associated with tularaemia seropositivity (Table 1). These are typical for several clinical forms of tularaemia. However, tularaemia was not clinically diagnosed in any of the patients.

## Discussion and conclusions

Considering that tularaemia had not been reported in Kenya before, the seroprevalence was surprisingly high. Despite this, these infections were not recognized by the local clinicians during hospital diagnosis. A similar diagnostic approach was applied in a previous study, revealing a sensitivity of nearly 100% and specificity >99% for the detection of tularaemia in humans [22]. In addition to the case report from Sudan [11], this study further suggests that tularaemia may be present in eastern Africa, but remains unreported. Therefore, regional rather than national efforts for an integrated approach to a sustainable surveillance system for tularaemia are needed. This is particularly necessary considering the ongoing security threat across the region and the potential use of *Francisella* in warfare or bioterrorism.

Communities in northern Kenya are predominantly pastoralists. Their nomadic movements lead them into different ecosystems and bring them into contact with wildlife and different vectors [26]. Contact with rodents that can be a reservoir of *F. tularensis* might play a role in the transmission of the disease, but the use of surface water as the main source of drinking water for wild animals, livestock and humans in the study region may be the most important reason for human infections. Further research is necessary to identify local vectors and reservoirs, and to identify key risk factors for infection.

This report emphasizes the need for local clinicians to broaden their diagnostic repertoire for undifferentiated fevers. This strategy would be of clinical importance as recent studies showed that clinicians in Kenya continue to treat febrile patients for presumptive malaria or use β-lactams, which are ineffective against *F. tularensis*, *C. burnetii*, *Yersinia* spp. or *Brucella* spp. A limitation of this study is that microbiological isolation of *F. tularensis* was not performed. Hence, the possibility of cross-reactivity with antibodies of other Gram-negative bacteria cannot be ruled out.

Another potential limitation is that the study used acute-phase serum samples only. Follow up of patients to obtain a convalescent serum sample was not feasible because of ongoing inter-clan conflicts and militia activities in the region. Therefore it was not possible to demonstrate seroconversion or a four-fold titre rise of anti-*Francisella* antibodies to definitively confirm tularaemia diagnoses in our patients. More research is needed to prove our findings using cultivation and further characterization of the bacteria.
